# Frost formation through super-cooled water within micron gap of galvanic coupled arrays

**DOI:** 10.1039/d2ra04217g

**Published:** 2022-12-02

**Authors:** Moataz Mekawy, Ken Hirayama, Yukihiro Sakamoto, Jin Kawakita

**Affiliations:** Electric and Electronic Materials Fields, Electrochemical Sensors Group, Research Centre for Functional Materials, National Institute for Materials Science 1-1 Namiki Tsukuba 305-0044 Japan KAWAKITA.Jin@nims.go.jp; Chiba Institute of Technology 2-17-1, Tsudanuma Chiba 275-0016 Japan

## Abstract

Frost is considered one of the key factors that negatively affects numerous daily life aspects all over the globe such as growth of crops, safety of aviation and transportation vehicles, working efficiency of air circulating systems and many others. Therefore, monitoring and early detection of frost are crucially needed to avoid such drastic effects. In this study, we used the micron gap of our newly developed galvanic coupled arrays named as moisture sensor chip (MSC) for the early detection of frost formation from super-cooled water droplets. The early frost formation was monitored *via* the tiny ice crystals formed on the cooled MSC surface at four different humidity levels using simultaneous electrochemical and optical microscopic detection tools. Experimental results revealed for the first time a remarkable increase in the detected galvanic current due to the condensation frosting mechanism of super-cooled water droplets *via* liquid transition transformation even at very low relative humidity which was believed to be responsible for de-sublimation frosting. Moreover, the super-cooled droplets formed ice bridges along their boundary domains due to the accumulation of the acquired water vapour that was evidenced by the release of the heat of solidification. These findings demonstrated that the MSC could be used as a promising platform for the early detection of frost formation considering the appropriate protective measures against its adverse effects.

## Abstract 2

Detection of frost formation through super-cooled water within the micron gap of galvanic coupled arrays.

## Graphical abstract

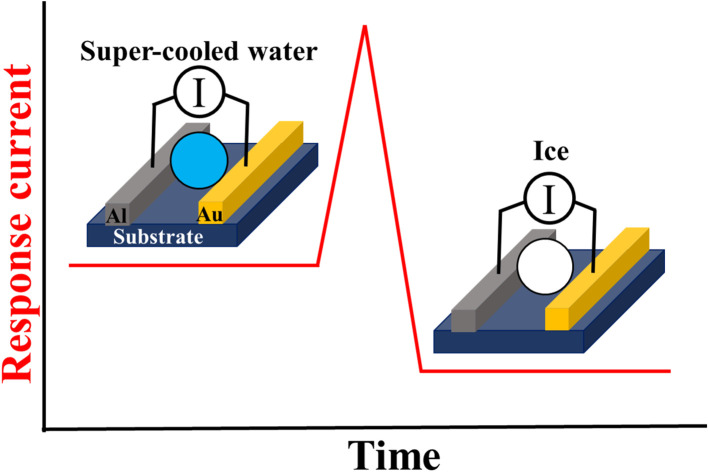

## Introduction

Frost can be defined as a specific weather event that can influence daily life. Frost-accumulating weather events have been remarkably increasing in recent years causing severe damage of several mankind aspects. For instance, significant reduction in crop yields which in turn leads to severe economic loss in addition to insecure nutrition strategies to meet with daily increased demands,^1–3^ inefficient air flow of turbines that are attached to aircraft and transportation vehicles subjecting them to crashing and threatening passengers' lives,^4^ and inefficient working or weakening of freezing, heating, ventilating and air conditioning systems which leads to serious economical waste of energy.^5^ To avoid or even minimize these adverse effects, early frost detection should be employed accurately along with appropriate supporting technologies.^6^

In principle, there are several types of frost.^7^ (I) Advection frost which has a form of ice spikes and forms when the target solid surface is subjected to a cold wind. (II) Window frost which is formed on single glass windows mostly during the winter season at cold places due to the temperature difference between inside and outside. (III) Rime frost which has a form of solid ice and is formed rapidly during very cold, wet and windy weather on the solid target surface. (IV) Radiation frost which is the most common frost type and is sub-divided into hoar and black frost. A relatively heavy coating of hoar frost forms white frost; however, in black frosts, there is no surface ice due to very low and negative dew points.^8^ Thus, white frost usually takes the form of tiny ice crystals that are formed when air is cooled to a saturation or dew point below freezing. The water vapour in the air then condenses in the form of ice crystals onto exposed solid surfaces. If the temperature is more than 0 °C, then dew will form. Subsequently, frost formation is inevitable once a cold solid surface at a temperature lower than that of the water triple point and air dew point is subjected to moist air. Hence, the dew or saturation point is defined as the temperature at which air can no longer hold any more moisture and the water vapour in the air condenses onto exposed solid surfaces. As a result, the formation of frost occurs when the temperature of the solid target surface drops below the water triple point enabling the freezing of water droplets to occur from the atmospheric state where dew is generated and the water vapour in the atmosphere sublimates or condenses enhancing frost growth.^9^ Since frost contains numerous aggregated types of ice crystals, then, we use frost as a general word throughout this paper.^10^

The mechanism of frost formation can be classified into two types:^11^ one occurs directly from the vapour phase through de-sublimation (de-sublimation frosting) and the other occurs *via* water vapour condensation on cooled solid surfaces creating a transient condensate of super-cooled water droplets that can be frozen through nucleation (condensation frosting). Both types are considered to depend on the structure and the temperature of the solid target surface along with its surrounding water vapour pressure expressed as relative or absolute humidity. Absolute humidity is believed to play an effective role in frost formation. Compared to the environment where condensation frosting occurs, de-sublimation frosting occurs at low absolute humidity of about 2.3–2.8 g m^−3^, and condensation frosting occurs at higher absolute humidity of minimum 3.2–3.8 g m^−3^.^12^ However, concrete experimental evidence is not clearly elucidated. Therefore, there is a crucial demand for sensitive and accurate early detection of frost formation taking into account the formation conditions and mechanisms. Such aim is believed to enhance frost formation understanding providing protective and appropriate anti-frost measures before its occurrence.

Since frost growth is accompanied by inevitable economic losses such as damage of crops, roads and many others,^12,13^ numerous studies on frost damage have been conducted. However, actual micrometeorological measurements prior to or during the time of frost damage at a certain location with a small topography remain uncertain. This could be due to the changes and differences in ground cover conditions. Nevertheless, it is still difficult to predict the date of occurrence.^13^

So far, several detection tools have been used for early frost formation. For instance, Salisbury *et al.* used a thermal infrared spectrometer to detect the reflectance spectra of feather-like frost crystals and wrapped frost crystals to obtain true frost reflectance.^15^ Wiltshire *et al.* used a planar double split ring resonator sensor to monitor frost formation on the sensor's surface at atmospheric pressure and ambient relative humidity of 21% by gradually chilling the sensor from 23 °C to −10 °C at a rate of 0.2 °C per second. The sensor was able to monitor the water-to-ice and ice-to-water phase changes in a single droplet with a variant resonant profile, including resonant amplitude, resonant frequency and the quality factor.^16^ As a result, it was found that there are several important parameters that may affect frost growth such as air relative humidity, super-cooling degree, and flow rate.^17,18^ However, to date, the frosting formation process within the micron gaps of galvanic coupled arrays has been neither examined nor reported.

Our previous research demonstrated the fabrication of a new unique hydrophilic micro-patterned sensor platform consisting of a micron gap within its galvanic coupled arrays denoted briefly as moisture sensor chip (MSC). MSC is composed of a repeated comb-like structure from two dissimilar Au and Al metal arrays interspaced with a confined SiO_2_ surface that possesses a tailored gap size varying between 0.5 and 10 μm. It was successfully used as a moisture sensor employing the dew condensation arising when a water droplet adheres on its surface bridging between the Au and Al arrays. As a result, a response galvanic current could be rapidly detected with excellent sensitivity and reproducibility.^19^ This unique feature enabled us to use MSC to detect a tiny amount of water droplets based on their size.^20^ Hence, we could establish a new feasible methodology for the quantification of condensed water droplets on the MSC surface based on the response current and image processing techniques.^21,22^ Moreover, we used electrochemical, gravimetric, and spectroscopic techniques to emphasize the response electrical properties, mass and nature of stacked water molecules within the interface of the MSC under a systematic RH controlled scheme.^23^ This enabled us to have a wide detection range of MSC that can distinguish between condensed water droplets and adsorbed water molecules.^24^ In addition, enhancing MSC's practical applicability using modification of its surface according to the heat capacity of the actual target object was carried out and the MSC output response showed a clear dependence on the variation in the cooling rate, as well as the vapour pressure.^25,26^ However, the humidity-based detection of frost formation at the surface of hydrophilic MSC was neither examined nor reported.

In this study, we monitored the frost formation from a water vapour stream condensate on cold MSC substrate in a controlled humidity environment using simultaneous microscopic and electrochemical measurements. In other words, we monitored the frost formation under various humidity levels where the MSC substrate was cooled in the range of 20 to −25 °C. Our experiments were carried out at fixed cooling rate of 15 °C min^−1^. Therefore, the dew temperature and the number of occupying water/ice molecules could be estimated.

## Experimental

### Fabrication of MSC

**Fig. 1:**
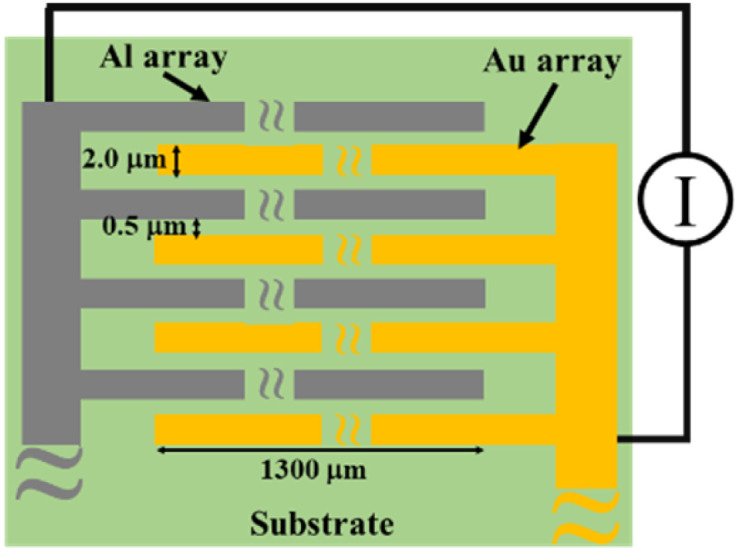
A schematic illustration of MSC surface.

MSC was fabricated as previously described.^19,25^ Briefly, a Si wafer was used as a substrate on which a silica layer was formed by thermal oxidation treatment. Au and Al arrays with length, width and height of 1300 μm, 2.0 μm and 0.2 μm, respectively, were alternately arranged on the substrate to form an opposed comb-like structure with 0.5 μm array interval. The number of Au and Al array pairs was 92. In addition, two Pt resistors of 10 μm width, 1000 μm length and 100 nm thickness were mounted at the edge of the MSC surface to monitor the temperature change with experimental duration. Afterwards, the wafer was cut into 5 mm^2^ each and the MSC was glued on an aluminium lead frame. Except for the array part, the remaining surface including the Pt resistance and wiring was covered with a polymeric resin to make a sensor package. Fig. 1 shows a schematic illustration of the MSC that was used in this study. The sensor package is attached to an electric circuit board by a connecting adapter and the electric current between two Al and Au arrays was measured using a tailored device with a 20 bit, octal channel, current-input analogue-to-digital (A/D) converter (DDC118, Texas Instruments) installed on the electric circuit board. The temperature of the MSC surface was estimated from the calibrated straight line of electrical resistance generated from Pt wires located on its surface as a function of changing temperature.^20^ Whenever a water droplet bridges between two arrays as a function of dew point condensation, the response galvanic current could be detected on the MSC surface with excellent sensitivity and reproducibility and with a minimum response time of 20 ms.

### Experimental setup

**Fig. 2:**
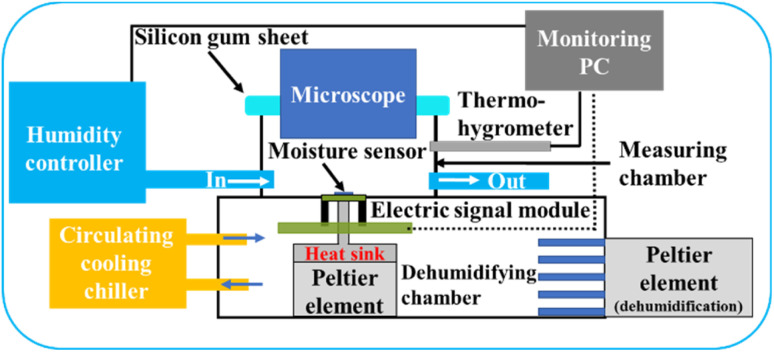
A schematic illustration of the experimental setup used for frost monitoring at cooled MSC surface using microscopic water droplets in a humidity-controlled scheme.

The experimental setup was established as shown in Fig. 2. In a typical experiment, the MSC/module set was mounted in a sealed measurement chamber. The internal temperature of the chamber was controlled and cooled below the water freezing point by using a built-in Peltier element. A metal thermal conductor working as a heat sink was attached to the Peltier element and cooled from the back of the MSC to enable the control of its surface temperature. A precise humidity control generator (Micro Equipment Inc., me-40DPRT-2FM-MFC) was used to set the measurement chamber relative humidity. A cooling circulating chiller was used to maintain the provision of super-cooled water droplets to the cooled MSC surface and to preserve the experimental conditions. Therefore, the water vapour pressure inside the chamber could be adjusted to the desired relative humidity allowing dew condensation to occur and enhancing the frost formation on the cooled MSC solid surface. A dehumidification chamber was used to regulate the desired humidity during the experimental time employing another Peltier element. The relative humidity and temperature inside the measurement chamber were measured using a thermo-hygrometer (EE23; E+E Elektronik, Engerwitzdorf) with ±1.3% RH accuracy for relative humidity and ±0.25 °C accuracy for temperature. The response current was measured at 0.1 s intervals. In addition, a high-speed optical microscope (VW-600C, KEYENCE Ltd, Japan) located above the measurement chamber was used to monitor the MSC surface simultaneously along with its monitored galvanic current response.

### Effect of temperature excursion

In this experimental scheme, the measurement chamber humidity was adjusted to 15% RH. Cooling of the MSC surface was carried out using its connected Peltier element. The starting temperature was 20 °C which represents the surrounding environment and the Peltier element set temperature was varied from −10 to −25 °C. The frost formation process was monitored *via* recording response galvanic current and using optical microscopy at each examined case. In other words, the frost formation process was monitored on the cooled MSC surface while regulating its surface temperature between 20 °C to a designated Peltier set temperature varying from −10 to −25 °C.

### Effect of humidity of the gas stream

Using the same aforementioned experimental setup, the precise humidity control generator was used to set the measurement chamber relative humidity between 12 and 53% with air at 200 NCCM flow rate. For minimum humidity level, N_2_ gas was supplied into the measurement chamber at 1000 NCCM.

Table 1 lists the conditions inside the measurement chamber that were used during the detection of frost formation experiments. At each examined humidity, the electrochemical response galvanic current was recorded. Moreover, the frost formation process on the cooled MSC surface was monitored by optical microscopy.

**Table 1:** The measurement parameters used in the measurement chamber during the detection of frost formation

Chamber temperature (°C)	Relative humidity (%)	Absolute humidity (g m^−3^)
22.3	5.2	1.1
22.2	12.0	2.4
22.4	33.6	7.2
22.5	53.4	10.7

## Results and discussion

### Frost detection under temperature excursion effect

**Fig. 3:**
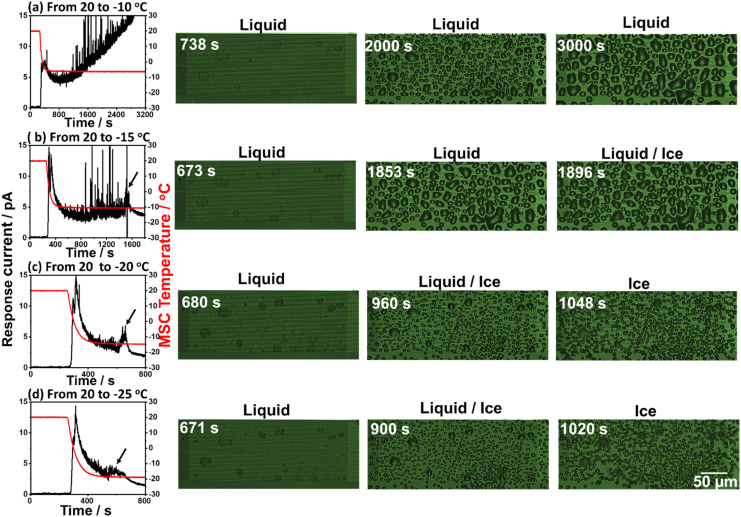
Simultaneously detected galvanic response current and microscopic images during frost formation process on the surface of MSC at set temperatures of −10 °C (a), −15 °C (b), −20 °C (c) and −25 °C (d). Scale bar is 50 μm.

In this experimental scheme, frost formation was monitored at a fixed relative humidity of 15% while preserving the starting temperature at 20 °C and varying the Peltier element set temperature between −10 and −25 °C. In each case, the response galvanic current and microscopic images were recorded. Fig. 3 shows the simultaneously recorded response galvanic current and microscopic images of the MSC surface as a function of time. Experimental results revealed the existence of a response current peak at around 370 s for all examined set temperatures. This current is due to the dew condensation of water droplets between the Al and Au arrays located on the MSC surface. Further response current peaks were not detected at −10 °C set temperature indicating that frost formation under these conditions may occur if the experimental time is extended to longer than 3200 s. However, additional current peaks (indicated with arrows) were recorded at other set temperatures followed by a decrease to their minimum steady values. These current peaks are attributed to condensation frosting formation (explained in the next section). Moreover, the time required to achieve complete frost formation became shorter once the set temperature was reduced. In addition, the MSC surface temperature showed a decreasing tendency with the set temperature. Thus, the frost formation process was monitored on the MSC surface at 0.5 μm gap size between Al and Au arrays before (liquid) and at the beginning of frost formation (liquid/ice) and at complete solidification (ice) from water droplets to frost. These results suggested that lower set temperature is more favourable and applicable for the clear investigation of frost formation. This could be attributed to the earlier occurrence of dew condensation along with the existence of a wider temperature range which enables explicit examination of the frost formation process. It should be noted that current noises remained notable at −10 and −15 °C set temperatures even after employing a moving average analysis method. This might be ascribed either to the dynamic coalescence behaviour of small water droplets to form bigger ones or to normal noises.

### Frost detection under controlled humidity

**Fig. 4:**
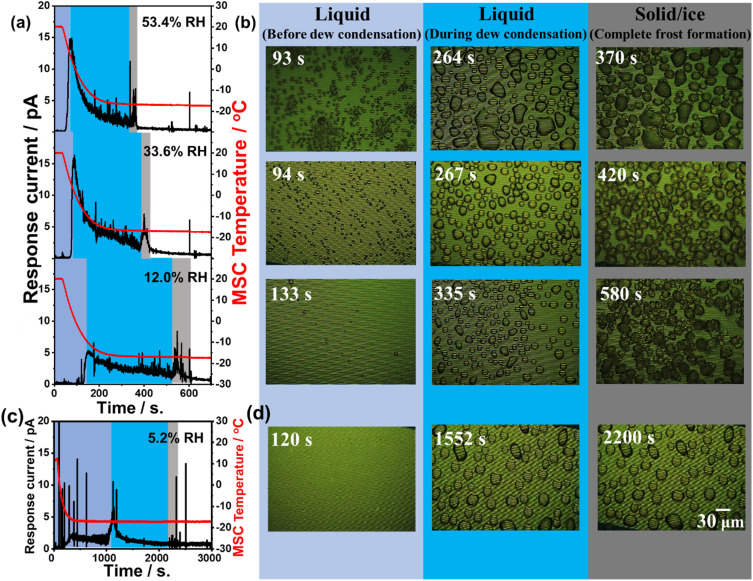
Time-dependent response galvanic current and the MSC temperature recorded at 12%, 33.6% and 53.4% RH (a), 5.2% RH (c) along with the monitored microscopic images (b and d) before (light blue) and during dew condensation (blue) and after complete solidification (grey) of microscopic water droplets. Scale bar is 30 μm.

Fig. 4(a) and (c) presents the time-dependent change in response current along with the MSC surface temperature monitored at various examined relative humidities of 5.2%, 12.0%, 36.4%, and 53.4%. Experimental results revealed that there are three different regions. The first region (light blue colour zone) was where the super-cooled water droplets were in a stage before dew condensation. The response current in this region was remarkably small at all examined relative humidity conditions. The second region (blue colour zone) was where water droplets started their dew condensation on the cooled MSC surface. This was remarkably evidenced through the increase in the response galvanic current. This galvanic current started to decrease again indicating lower droplet conductivity and a preliminary frost formation within water droplets. Both regions concern the range where water droplet temperature is below the freezing point. The third region (grey colour zone) was where a sudden increase in the galvanic current was notably detected followed by further decrease reaching its lowest steady state indicating complete freezing. These results suggested the existence of a phase change towards the formation of liquid water droplets that was followed by a complete formation of solidified frost phase at the freezing point. Thus, the third region represents the time at which the water droplets on the MSC surface were completely solidified. Results also demonstrated that once the % RH increases, the time required to reach complete solidification becomes shorter. Moreover, the response current near the third region is higher than that at the second region. These results also indicated that low relative humidity conditions lead to a slow dew condensation behaviour that affects increasing the time required to reach the frost phase. In addition, Fig. 4(b) and (d) presents the microscopic images of the MSC surface recorded simultaneously. Images highlighted in light blue represent the time at which the MSC surface was below the dew condensation and the water freezing point. The shape of water droplets at this stage was somehow randomly oriented having a small area. Images highlighted in blue represent the time at which the water droplets grew (during dew condensation) to well-recognized hemispherical shapes with larger areas and started to solidify on the MSC surface. Finally, images highlighted in grey represent the time at which the hemispherical water droplets were completely solidified forming frost on the MSC surface whose temperature remained fixed at about −17.5 °C. A previous report demonstrated that the sensor response was relatively increasing once the area of water droplets was increasing.^27^ However, our results indicated that there was a decrease in the detected galvanic current response under the examined relative humidity conditions.

Taking into account the real detection fields where the water droplets are in a tiny microscopic area and/or volume similar to that in our experimental setup, one can predict that the diameter of the water droplets on the MSC surface will be much less than 10 μm (average volume was around 0.3 pL assuming a hemispherical shape). The detected response galvanic current between Al and Au arrays was smaller than that detected with larger mesoscopic volume used for direct water droplet dropping (explained in mesoscopic detection section). As a result, the total amount of solidification heat generated in the solidification process was also smaller indicating that the temperature change of the entire MSC surface was below the lower limit of detection. Moreover, Fig. 4 shows the water droplets that were gradually and individually solidified reaching finally a complete solidification of all water droplets on the MSC surface. These results suggested that when super-cooled water droplets solidify on the MSC surface, some water droplets start to coalesce together. In addition, it is considered that the detected spike-like response current is due to the sudden increase and decrease in the response galvanic current arising from the water droplets accompanied by a release of heat of solidification. However, the change in recorded MSC temperature was not remarkable. This could be ascribed to the tiny microscopic amount of water droplets that transformed to frost generating a minute amount of undetectable heat of solidification. Nevertheless, the MSC temperature was recorded *via* Pt thermocouples that were located at MSC edges that might be little far from the real core reaction position. To investigate these findings, further experimental work was carried out examining water droplets of larger volume (Frost detection from mesoscopic water droplet section). As evidenced from Fig. 4(a), a spike-like increase and decrease of the response current in a short time was observed. The number of these spike-like responses was relatively notable in the third (grey) region. As shown in Fig. 4, this is considered to be the change in current due to the coalescence of water droplets. These results revealed that when small water droplets coalesce, a larger hemispherical water droplet will be generated and consequently the area covered between the Al and Au arrays will be also increased temporarily followed by further shrinking reaching a steady state. It is also considered that the current fluctuations due to the arrays will be also increased temporarily followed by further shrinking reaching a steady state. It is also considered that the current fluctuates due to the internal convection generated when the water droplets coalesce. These findings suggested that the frost formation on our MSC occurred *via* the condensation frosting mechanism. In principle, condensation frosting is defined as an indirect way of frost formation where water vapour condenses into super-cooled liquid that freezes later into ice. In other words, super-cooled droplets freeze in isolation by heterogeneous nucleation at the solid–liquid interface. However, de-sublimation is defined as the direct way of frost formation where water vapour transforms directly into ice without passing through the liquid phase.

Previous research work suggested that the success and rate of inter-droplet frost growth are dependent mainly on two factors: first, the extent of spacing between hydrophilic regions where liquid nucleation occurs; second, the time required for condensation growth before the initial freezing event.^28^

Taking into account the unique composition features of our developed MSC, possessing a hydrophilic ternary-texture structure of Au and Al arrays that are interspaced with a confined area of SiO_2_ acting as a wedged-shape pocket, one can predict that this micro-structured surface can be used efficiently to control droplet distribution as a frost condenser. Our results of the freezing of condensed super-cooled water droplets on a cooled solid MSC surface demonstrated that the vapour pressure condensate affects the formation of water droplet bridge connections, enhancing the formation of larger frost grains that can be enlarged to form a full frost film once the dew condensation continues. Furthermore, the microscopic images suggested that the water droplets on the MSC surface were completely solidified to frost as clearly evidenced by the stable response current values recorded after the second region. Therefore, it is expected that there was no more release of heat of solidification (no convection) taking place within the water droplet.

### Microscopic frost formation behaviour

**Fig. 5:**
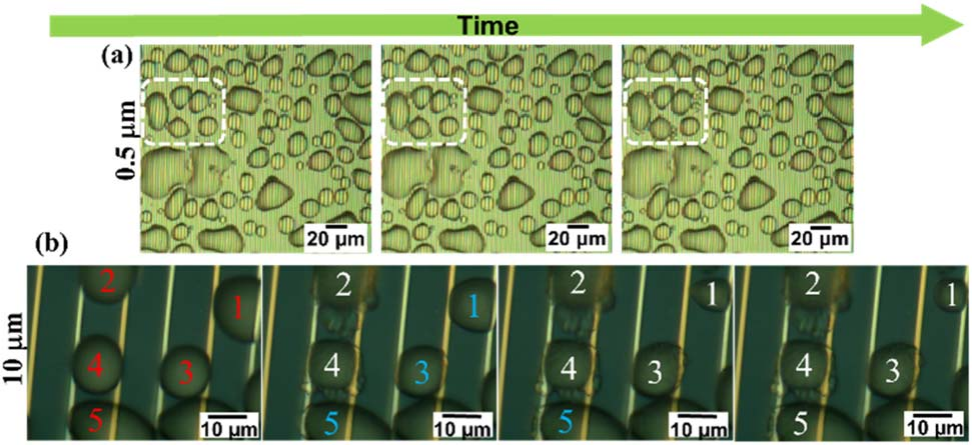
Dynamic formation of ice from water droplets *via* condensation mechanism using MSC gap size of 0.5 μm (a) and 10 μm (b), respectively. Red numbers are for liquid water droplets, light blue numbers are for partially frozen water droplets and white numbers are for frozen water droplets.

Careful monitoring of microscopic images was carried out and the experimental results obtained using a 0.5 μm gap size are shown in Fig. 5(a). Selected region demonstrated the freezing process while bridging neighbour droplets *via* hoar frost which was formed along a droplet's boundary. However, for better understanding of these results, we examined the same behaviour at the MSC surface with a 10 μm gap size. Experimental results shown in Fig. 5(b) evidenced that water droplet 1 was partially shrinking *via* evaporation with time. This evaporation was captured by the nearby droplets 2 and 3 to enhance their volume/area leading to the formation of hoar frost. Edges of this hoar frost were used to bridge with other droplets 4 and 5 in a preliminary successive formation of larger frost entity. This process continues to form a complete frost film.^29^ These results are fully matching with previously reported ones^30^ where liquid water droplets were feeding ice crystals by providing them with water vapour during their shrinkage and/or evaporation allowing ice crystals to grow. This growth mechanism is accompanied by a phase transition process in super-cooled clouds denoted as the Wagener–Bergeron–Findeisen (WBF) process. Thus, the stages of frost formation can be explained as: (i) condensation of super-cooled water droplets, (ii) solidification of isolated droplets and (iii) frost spreading bridging the solidified droplets to form a continuous film.

**Fig. 6:**
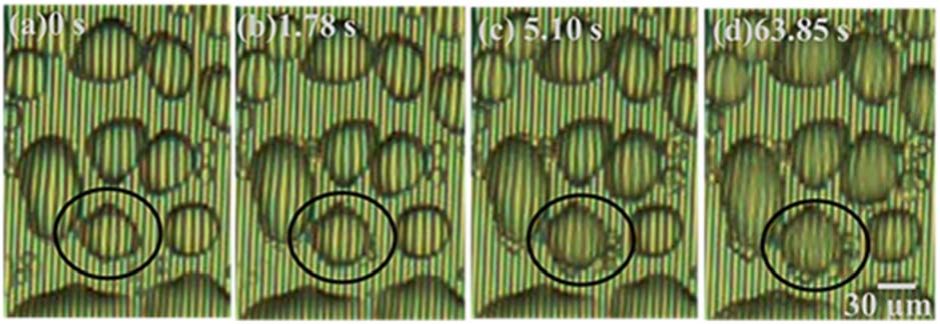
Real time-dependent microscopic images revealing the different stages of ice formation from super-cooled water droplets. Dew droplet formation (a), coalescence around water droplet (b), partial (c) and complete solidification stages (d), respectively.

Fig. 6 shows microscopic images of the detailed phase change of a selected droplet domain with time until reaching ice formation. Fig. 6(a) shows the early stage of droplet dew formation. Fig. 6(b) shows the starting of coalescence around the water droplet's outer surface. At that time, it is considered that the water droplet has not reached a solidification state due to the clear observation of arrays through the water droplet surface. Moreover, Fig. 6(c) shows that the minute water droplets were undergoing the solidification process and the arrays were difficult to be seen through the water droplet surface during this process. It is considered that the cooling surface of the minute water droplets began to solidify while the upper surface of the water droplets remained in a liquid phase. In addition, Fig. 6(d) shows that the arrays were not visible through the minute water droplets suggesting that the water droplets were completely solidified.

### Microscopic detection of frost formation at low humidity

**Fig. 7:**
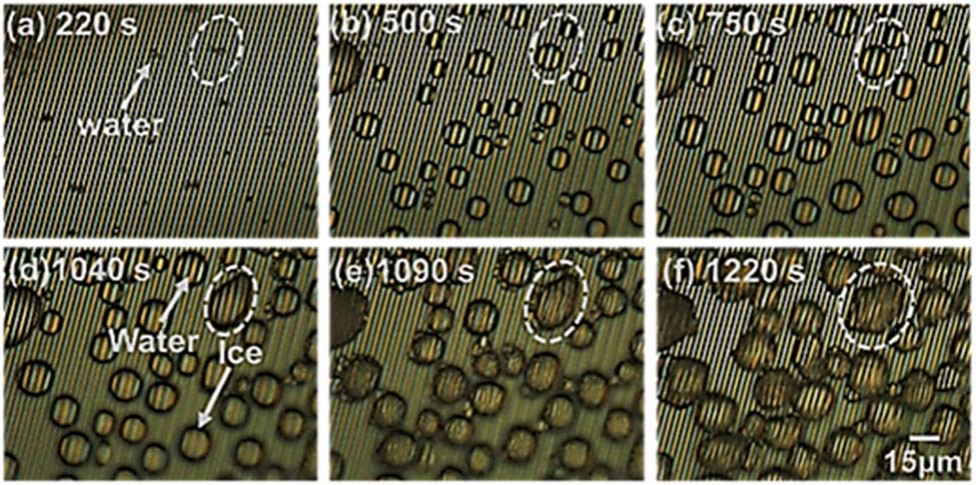
Microscopic monitoring of frost formation at 5.2% RH on the MSC surface that represents the early formation of dew condensed water droplets (a) followed by continuous growth to about 15 μm (b) and (c), partial solidification of some water droplets (d), complete solidification of all water droplets forming hoar frost around their surfaces and bridging with nearby droplets (e) and frost coalescence growth to bigger sizes on the MSC surface (f). Scale bar is 15 μm.

Further examinations were carried out at quite low relative humidity of 5.2% to monitor the early and stepwise formation of frost formation at the cooled MSC solid surface. Fig. 7 presents the detailed recorded time-dependent microscopic images. Fig. 7(a) shows the start of dew condensation from minute water droplets on the MSC surface. Fig. 7(b) and (c) shows the dynamical growth of water droplets to an average size of about 15 μm on the MSC surface. Fig. 7(d) shows that some droplets started to solidify. Hence, both water droplets and ice crystals were present on the MSC surface. Fig. 7(e) shows that water droplets were completely solidified; meanwhile, some droplets were partially or totally evaporated. This evaporation was exhibited by other water droplets forming hoar frost around their boundaries. These hoar tags started to bridge with other frozen droplets allowing them to coalesce. Fig. 7(f) shows the coalescence of water droplets to form larger domains of frost. This process is speculated to be continued until the formation of a full frost film on the MSC surface. These results confirmed that water vapour did not de-sublimate directly from vapour to form frost even at absolute humidity lower than 2.3–2.8 g m^−3^ which is the mandatory condition for frost formation. In contrast, water vapour transforms into transient liquid phase prior to the formation of solid-phase frost *via* the condensation mechanism.

**Fig. 8:**
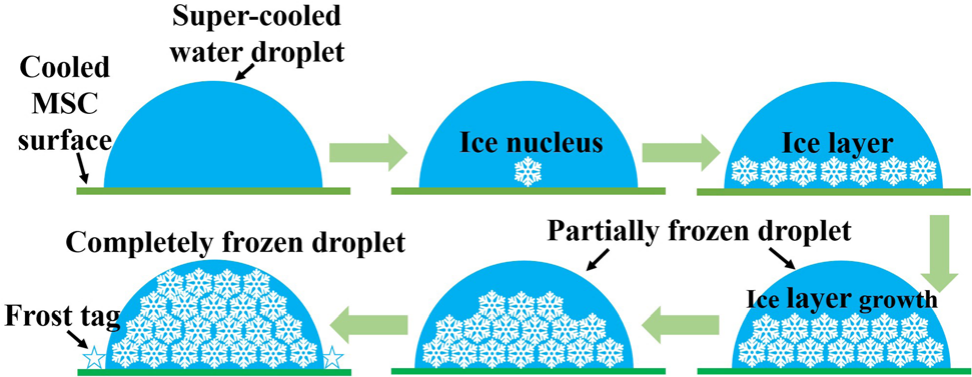
A schematic diagram of the solidification model of a small water droplet during the frost formation process on the MSC surface.

Fig. 8 shows a schematic illustration for the coalescence process in a small water droplet (diameter of 30 μm). As evidenced from Fig. 7(e), the coalescence starts around the water droplet's surface boundary. Meanwhile, it is considered that the water droplet itself was not solidified because it looks unchanged on the arrays that can be observed through the water droplet. In this case of minute water droplets (diameter = 30 μm), the edge of the water droplet in contact with the cooling surface solidifies, and the ice generated by de-sublimation from water vapour adheres and grows on it, causing the temperature around the edge to drop and that far from the edge to rise. It is presumed that the inside solidified in the lateral direction and then solidified vertically. Therefore, the solidification mechanism was as follows. A super-cooled water droplet adheres on the MSC cooled surface. Once the dew conditions are achieved, ice nucleus starts to form within the inner core of the super-cooled water droplet which is in close contact with the cooled MSC surface. Numerous ice nuclei are formed and nucleated together to form larger ice entities until reaching full cluster *via* lateral growth. Upper ice crystals function as new frost nucleation sites which in turn leads to the formation of further building layers *via* vertical growth. This process continues until the full solidification of the water droplet. These results suggested that the super-cooling time is inversely proportional to the volume of the water droplet as previously reported.^31^ Therefore, it is considered that the coalescence time became faster as the humidity increased.

### Frost detection from mesoscopic water droplet

In a control experimental scheme, a 40 μL pure water droplet was directly dropped on the cooled MSC surface. The response galvanic current and the MSC surface temperature were monitored during the solidification frost formation process. Experimental results revealed that the surface temperature and current were nearly stable up to 100 s. Afterwards, a remarkable decrease in both values was recorded up to about 650 s. A sudden increase in both values was recorded at a duration of about 0.1–0.3 s followed by a further decrease. This could be ascribed to the release of a certain amount of heat of solidification where the water droplet changed its status from vapour phase to ice or solid phase *via* passing through a transient liquid phase.

**Fig. 9:**
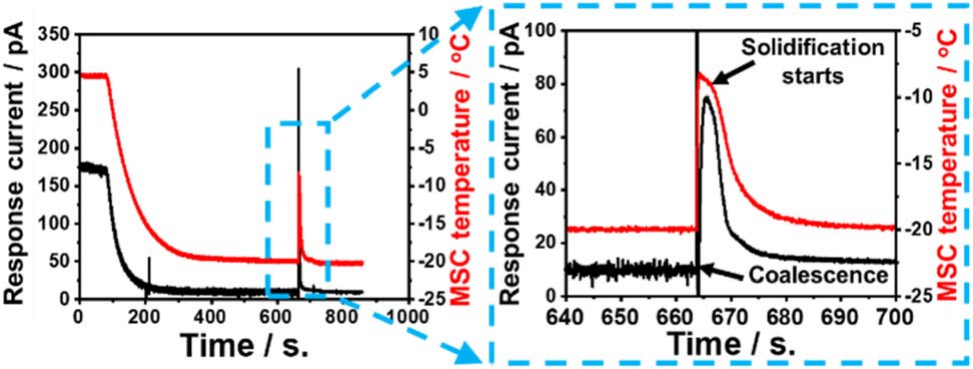
Time-dependent response galvanic current and surface temperature recorded during the direct dropping of a 40 mL water droplet on the MSC surface.

Fig. 9 shows the response current during solidification and the change over time in the sensor surface temperature when water droplets are dropped directly on the MSC surface. As the MSC surface temperature decreases, the response current also decreases. It is known that the conductivity of water decreases with temperature,^32^ and it is considered that the conductivity of water droplets in this study was also decreased and the response current was attenuated as the temperature decreased. Experimental results evidenced that there were notable rapid rise and fall in the response current and surface temperature just before the complete solidification of water droplets. It was reported that the temperature of super-cooled water droplets rises from the super-cooled state to the freezing point in a short time by eliminating super-cooling.^33^ Similarly, in our study, the temperature of the water droplets rises and the conductivity rises due to the heat of solidification generated when the super-cooled water droplets solidify on the MSC surface. The heat of solidification is defined as the heat released during phase transformation from liquid to solid at a certain preserved temperature. It is confirmed experimentally that the MSC surface temperature increased due to the heat released which is responsible for the increase in the detected response galvanic current. These results suggested that the MSC surface enhanced the catalytic formation of ice.

### Current and temperature responses upon solidification

Our experimental results revealed the existence of a transient liquid water phase due to the condensation frosting mechanism which in turn transformed into solid ice phase (grey regions in Fig. 4). This phenomenon was accompanied by a remarkable increase in the recorded temperature of MSC along with response galvanic current when large-volume droplets (*e.g.*, 40 μL) were employed, suggesting the existence of heat release as shown in Fig. 9. This heat was gained by ice and MSC leading, finally, to a significant temperature change. In contrast, tiny-volume droplets (*e.g.*, 40 nL) formed under controlled humidity levels did not show any remarkable temperature change despite recording an increase in the response galvanic current indicating that the heat involved was too small to induce a detectable change in temperature.

To explain these findings, we considered the system as approximately adiabatic, assuming that the sum of the heat released by the solidification of water and that absorbed by the ice formed and by the MSC (each taken with its own sign) was zero. We used Hess's law to estimate the total heat released during the solidification process (*Q*_t_) considering the following three consecutive transformations: (1) transformation from liquid water at −20 °C to liquid water at 0 °C, (2) transformation from liquid water at 0 °C to solid water (ice) at the same temperature, and (3) transformation from ice at 0 °C to ice at −20 °C. Each transformation is accompanied by a certain amount of heat denoted as *Q*_1_, *Q*_2_ and *Q*_3_, respectively. Accordingly:

1 *Q*_t_ = *Q*_1_ + *Q*_2_ + *Q*_3_

Therefore, eqn (1) can be rewritten as:2
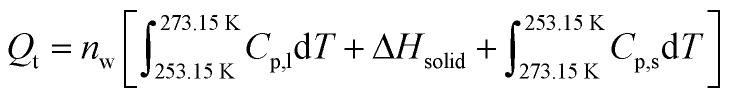
where *n*_w_ is the number of moles of liquid water in the droplets at each examined condition, *C*_p,l_ is the molar heat capacity of liquid water (75.37 J K^−1^ mol^−1^), *C*_p,s_ is the molar heat capacity of ice (37.84 J K^−1^ mol^−1^), and Δ*H*_solid_ is the molar heat of solidification (−Δ*H*_fusion_ = −6008 J mol^−1^).

This released heat (*Q*_t_) was then transferred to ice (*Q*_i_) that is located on the MSC surface and to the MSC itself (*Q*_s_). Therefore, the heat balance can be expressed as follows:

3 −*Q*_t_ = *Q*_i_ + *Q*_s_

Subsequently, *Q*_i_ can be calculated by the following equation:4
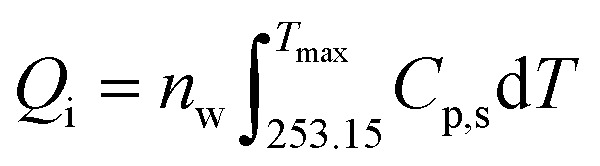
where *T*_max_ is the maximum detected temperature.

During the solidification process of a 40 μL (2.22 × 10^−3^ mol) liquid water droplet, a change in temperature from −20 °C to −8 °C was recorded. Thus, the total released heat, calculated according to eqn (2) (assuming the molar heat capacities of liquid and solid as constants) with *n*_w_ = 2.22 × 10^−3^, was ≈−10.66 J. Part of this heat was transferred to ice and could be estimated by eqn (4) considering *T*_max_ = 265.15 K (−8 °C), *i.e.* the maximum experimentally detected temperature. As a result, the estimated heat transferred to ice (*Q*_i_) during the solidification process was ≈1.00 J and that transferred to MSC was ≈9.66 J. This result allowed us to state that: (i) the heat absorbed by the ice is, to a first approximation, negligible compared to the heat transferred to the sensor; (ii) the thermal heat capacity of the sensor itself can be estimated as *C*_S_ = *Q*_S_/Δ*T* = 9.66/12 = 0.805 J K^−1^. The increase in conductivity of ice with temperature can easily justify the detection of response galvanic current. In the case of tiny water droplets, *n*_w_ could be estimated as previously mentioned.^22^ Briefly, the average volume of a single liquid water droplet was estimated directly before the solidification process and correlated to the whole number of droplets occupying a selected observed region.

Assuming a homogenous distribution of water droplets along the whole microscopic window region, the total number and the total volume of water droplets could be estimated and consequently *n*_w_ could be calculated, taking into account that the density of liquid water phase is 1 g cm^−3^. In a typical example, the total volume of water droplets at examined 33.6% relative humidity was estimated as 38.6 nL (*n*_w_ ≈ 2.14 × 10^−6^ mol). In this case, one can estimate the total released heat from tiny liquid water droplets during the solidification process as ≈−0.01 J. By considering the thermal heat capacity of the sensor as estimated above, the temperature change can be evaluated as Δ*T* = *Q*_S_/*C*_S_ ≈ 0.012 K. This temperature change is too small to be detected by temperature sensors of the MSC. However, it is reasonable to assume that, since the dispersion of minute droplets is a heterogeneous system, the achievement of thermal equilibrium with the MSC is not immediate; therefore, at least for a few seconds, the heat released in the formation of the ice crystals will be mostly absorbed by the ice itself, resulting in a local temperature change, which in turn leads to a significant change in the response galvanic current between the galvanic arrays. This experimental finding indicated that MSC can be beneficially used to detect explicitly the frost formation process based on the change in conductivity and consequently the current detection even from tiny-volume water droplets formed under controlled humidity levels.

The thermodynamical estimation of released heat together with experimental finding of response galvanic current increase confirmed that the formation of solidified frost on the MSC surface takes place preferentially *via* the condensation frosting mechanism while passing through a liquid transient phase and not *via* the de-sublimation mechanism where water vapor is directly transformed into ice solid phase.^4,14,34^

## Conclusion

To date, technologies that can be used to easily detect and reliably predict frost formation are quite rare. In this study, it was confirmed that the solid water phase named as frost was formed from the original phase of water vapour through the rapid formation of a transient liquid water phase due to the condensation frosting mechanism. This behaviour was confirmed even at low humidity (5.2% RH and 1.1 g m^−3^), which was believed to follow the direct de-sublimation mechanism. Possible detection of frost formation *via* electrical change was revealed for the first time. Our findings revealed that the response galvanic current detected from the MSC shows a characteristic behaviour with the heat released that was generated during the solidification process. Ice might be nucleated in the contact interface between a water droplet and the MSC solid surface enhancing lateral growth of an ice layer followed by further vertical growth until complete solidification. These findings suggested that our newly developed MSC could be feasibly applied for the real monitoring of frost formation. Further future applications could be expected in the field where it is necessary to suppress frost formation. Despite the simultaneous monitoring of frost formation by visual imaging techniques that are associated with controlled examinations of the environmental temperature and humidity, prediction and monitoring of frost formation in the open air and during nighttime are yet to be achieved to set the appropriate preventive measures against its adverse effects.

## Author contributions

M. M., Y. S., and J. K., conceptualization, results discussion and models formulation. K. H., experimental setup and data collection. Y. S. and J. K., research resources and project administration. M. M., data analysis and manuscript writing. All authors read, revised and agreed to the published version of the manuscript.

## Conflicts of interest

There are no conflicts to declare.

## Supplementary Material

## References

[cit1] Hunter M. C., Smith R. G., Schipanski M. E., Atwood L. W., Mortenson D. A. (2017). Bioscience.

[cit2] Jha K., Doshi A., Patel P., Shah M. (2019). Artif. Intell. Agric..

[cit3] Imam S. A. , Choudary A. and Sachan V. K., Int. Conf. Soft Comput. Tech. Implementations, 2015, pp. 181–187

[cit4] Petit J., Bonaccurso E. (2014). Langmuir.

[cit5] Yao Y., Zhao T. Y., Machado C., Feldman E., Patankar N. A., Park K.-C. (2020). Proc. Natl. Acad. Sci. U. S. A..

[cit6] Shammi S., Sohel F., Diepeveen D., Zander S., Jones M. J. K. Inf. Process. Agric..

[cit7] Kalma J. D. , Laughlin G. P., Caprio J. M. and Hamer P. J., The Bioclimatology of Frost, 1992, pp. 5–12

[cit8] Savage M. J. (2012). S. Afr. J. Plant Soil.

[cit9] Tkachev S. N., Nasimov R. M., Kalinin V. A. (1996). J. Chem. Phys..

[cit10] Borrebæk P.-O. A., Jelle B. P., Zhang Z. (2020). Sol. Energy Mater. Sol. Cells.

[cit11] Jung S., Tiwari M. K., Poulikakos D. (2012). Proc. Natl. Acad. Sci. U. S. A..

[cit12] Shibuya K. and Noborio K., JSSI & JSSE Joint Conference, 2017, vol. 9.24–9.27, p. 10

[cit13] Luo Y., Chen J. (2019). J. Traffic Transport. Eng..

[cit14] Drepper B., Bamps B., Gobin A., Orshoven J. V. (2021). Environ. Evid..

[cit15] Salisbury J. W., Aria D. M., Wald A. (1994). J. Geophys. Res.: Solid Earth.

[cit16] Wiltshire B., Mirshahidi K., Golovin K., Zarifi M. H. (2019). Sens. Actuators, B.

[cit17] Aguiar M. L., Gaspar P. D., Silva P. D., Silva A. P., Martinez A. M. (2020). Energy Rep..

[cit18] Hermes C. J. L., Piucco R. O., Barbosa Jr J. R., Melo C. (2009). Exp. Therm. Fluid Sci..

[cit19] Kawakita J., Chikyow T. (2017). ECS Trans..

[cit20] Kubota Y., Mishra V. L., Sakamoto Y., Kawakita J. (2020). Sens. Actuators, A.

[cit21] Terada E., Mekawy M., Sakamoto Y., Kawakita J. (2021). J. Electrochem. Soc..

[cit22] Mekawy M., Terada E., Inoue S., Sakamoto Y., Kawakita J. (2021). ACS Omega.

[cit23] Mekawy M., Noguchi H., Kawakita J. (2022). J. Colloid Interface Sci..

[cit24] Mekawy M., Terada E., Kawakita J. (2022). Chemosensors.

[cit25] Kubota Y., Satoh N., Mekawy M., Sakamoto Y., Kawakita J. (2021). J. Electrochem. Soc..

[cit26] Shrestha R. G., Kubota Y., Sakamoto Y., Kawakita J. (2020). Sensors.

[cit27] Asay D. B., Kim S. H. (2005). J. Phys. Chem. B.

[cit28] Boreyko J. B. (2016). et al.. Sci. Rep..

[cit29] Nath S., Ahmedi S. F., Boreyko J. B. (2017). Nanoscale Microscale Thermophys. Eng..

[cit30] Guadarrama-Cetina J., Mongruel A., Gonzalez-Vinas W., Beysens D. (2013). Europhys. Lett..

[cit31] Jung S., Tiwari M. K., Doan N. V., Poulikakos D. (2012). Nat. Commun..

[cit32] Marshall W. L. (1987). J. Chem. Eng. Data.

[cit33] Mossop S. C. (1957). Proc. Phys. Soc., London, Sect. B.

[cit34] Hauer L., Wong W. S. Y., Azadeh S.-A., Kondic L., Vollmer D. (2021). Phys. Rev. E.

